# Perineal pseudocontinent colostomy is safe and efficient technique for perineal reconstruction after abdominoperineal resection for rectal adenocarcinoma

**DOI:** 10.1186/s12893-015-0027-z

**Published:** 2015-04-10

**Authors:** Amine Souadka, Mohammed Anass Majbar, Tijani El Harroudi, Amine Benkabbou, Abdelilah Souadka

**Affiliations:** Surgical Department, National Institut of Oncology, University Mohammed Vth Souissi, Rabat, Medical School, Rabat, Morocco; Department of General Surgery, University Mohammed Vth Souissi, Rabat, Medical School, Rabat, Morocco; Surgical Department, Al Azhar Oncological Center, Rabat, Morocco

## Abstract

**Background:**

The aim of this study was to evaluate oncologic results and satisfaction rate results of pseudocontinent perineal colostomy (PCPC) using Schmidt’s technique in patients undergoing abdominoperineal resection (APR) for managing low rectal adenocarcinoma.

**Methods:**

From January 1993 and December 2007, One hundred and forty six patients underwent successfully PCPC after abdominoperineal resection for lower rectal adenocarcinoma. There were 75 women, with an average age of 47 years old. All patients received neoadjuvant radiotherapy with or without chemotherapy. Long-term oncological results and satisfaction rate were evaluated.

**Results:**

After a median follow up of 36 months (range 12–156) months, the five-year overall survival and disease free survival rate were 74.6% and 60.3% respectively. Local and distant recurrences occurred respectively in 10 (6,8%) and 29 (20%) patients. Seventy-seven percent (77%) of the patients were highly satisfied with this technique and only one patient was unsatisfied. However, none of them accepted the conversion to an abdominal colostomy neither would have changed PCPC for an iliac colostomy at first intent.

**Conclusion:**

This study showed that pseudocontinent perineal colostomy is a safe and reliable pelvic reconstruction technique after abdominoperineal resection for low rectal adenocarcinoma. It provides high degree of patient satisfaction without compromising oncological results. It is a good option in selected patients, especially in Muslim countries.

## Background

The management of ultra-low rectal cancer less than 2 cm from the dentate line is still challenging. The only two procedures available are an abdominoperineal resection (APR) with definitive iliac colostomy and intersphincteric resection (ISR) with a coloanal anastomosis. However ISR oncological and functional results are still debatable, the reason why APR remains the gold standard technique when both internal and external sphincter are involved.

Perineal pseudocontinent colostomy (PCPC) is a reconstruction technique performed after APR, in which the permanent colostomy is placed in the perineum instead of left low quadrant of the abdomen [1]. In this procedure, a graft of smooth colonic muscle tightly surrounds the lowered colon. This technique was first described by Schmidt [2], for abdominal colostomies, and then applied by Gamagami to the perineum [3]. It was encouraged by the fact that it offers two major advantages: preservation of the body image by invisible perineal placement, improving the quality of life of these patients [4], and a reasonable continence with acceptable functional results [3,5-7]. Since previous studies reported that abdominal colostomy is associated with a negative impact on the quality of life [8-10], especially in Muslim patients [11], this technique could be a very interesting for these patients [12].

The main concern about PCPC in all previous published studies was only its promising functional results [3-7,13-16]. Only Goere and al reported oncological outcomes of PCPC after APR for 19 anal epidermoid carcinoma.

The aim of this study was to evaluate long-term oncologic results and patients satisfaction of PCPC using Schmidt technique in Moroccan patients undergoing APR for managing lower rectal adenocarcinoma.

## Methods

From 1993 to 2007, 380 APRs for low rectal adenocarcinomas were performed in the Department of Surgical Oncology in both the National Institute of Oncology and the Al Azhar Oncological Center in Rabat, Morocco. Both surgical oncology departments of the institutions cited above granted permission to access data of included patients for the purpose of this study. The medical ethical comity of both institutions approved this study.

The design of this study, indications and functional results of patients with PCPC were already published in previous publication [7]. One hundred forty six patients underwent successful perineal reconstruction by PCPC.

### Surgical technique

**Figure 1:**
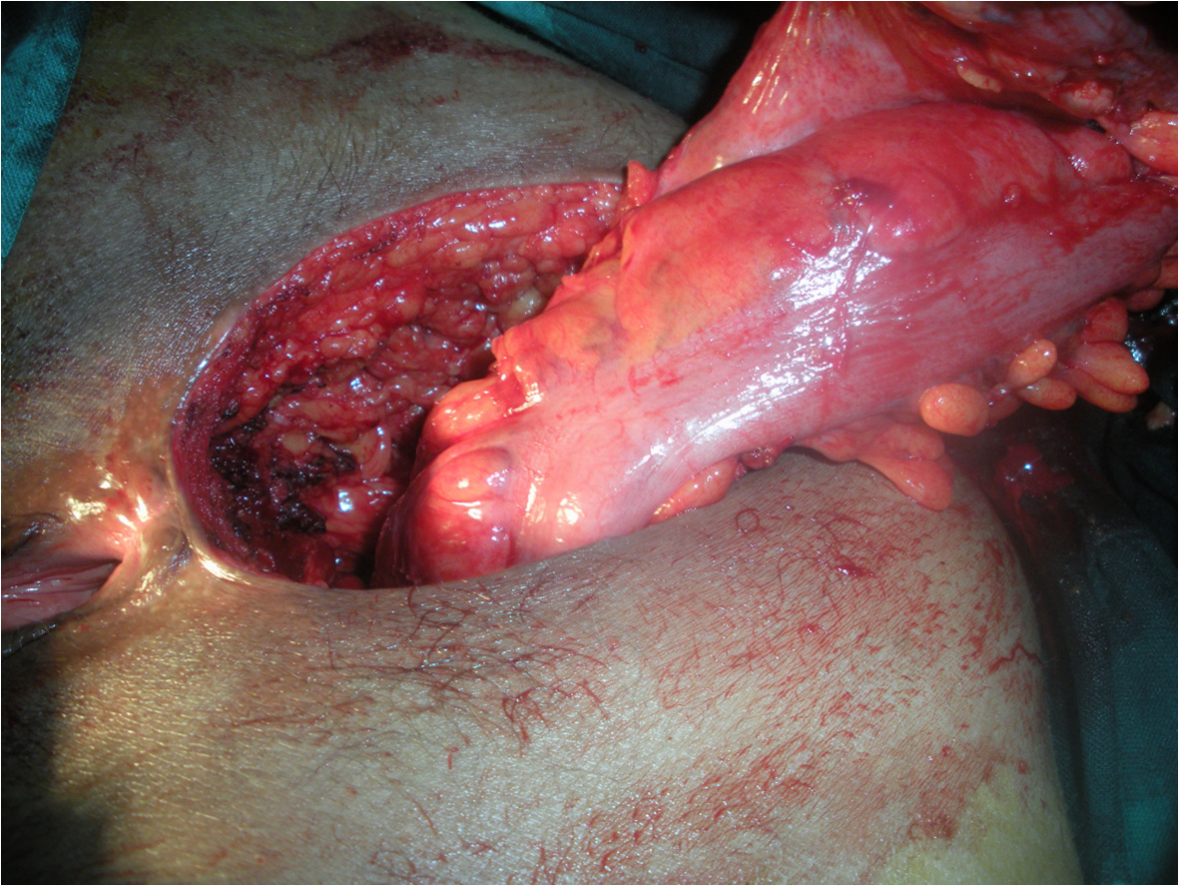
**Excision of the specimen by a perineal approach.**

**Figure 2:**
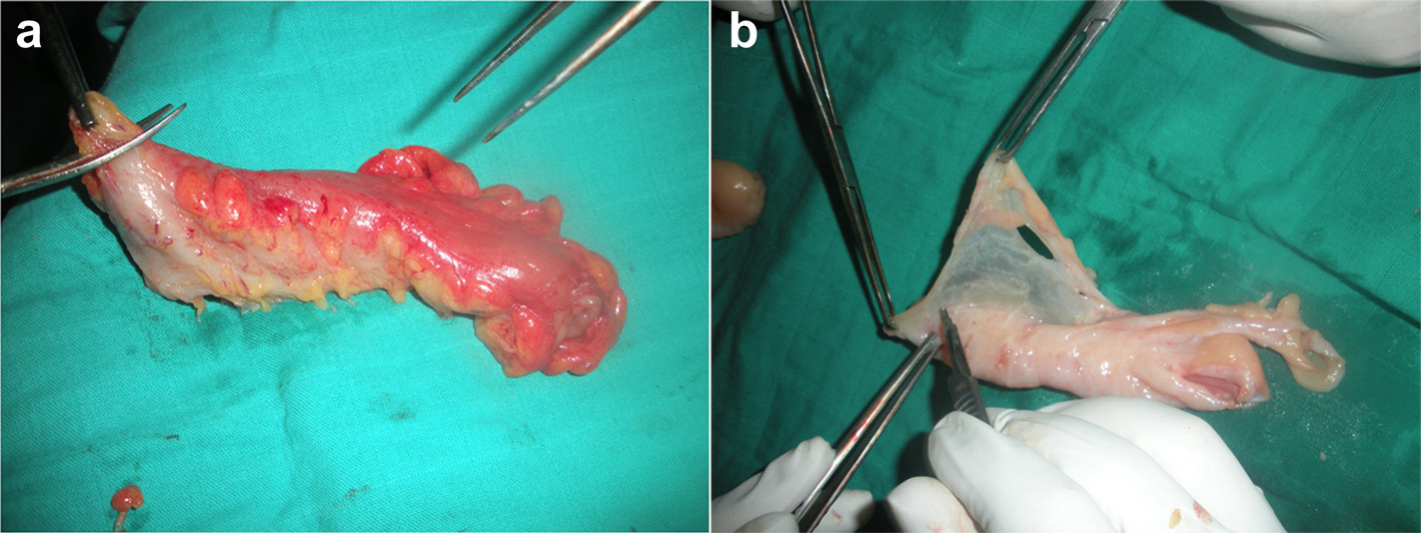
**Colic graft preparation. a**: Stripping from its meso and epiploics. **b**: Stripping from its mucosa.

**Figure 3:**
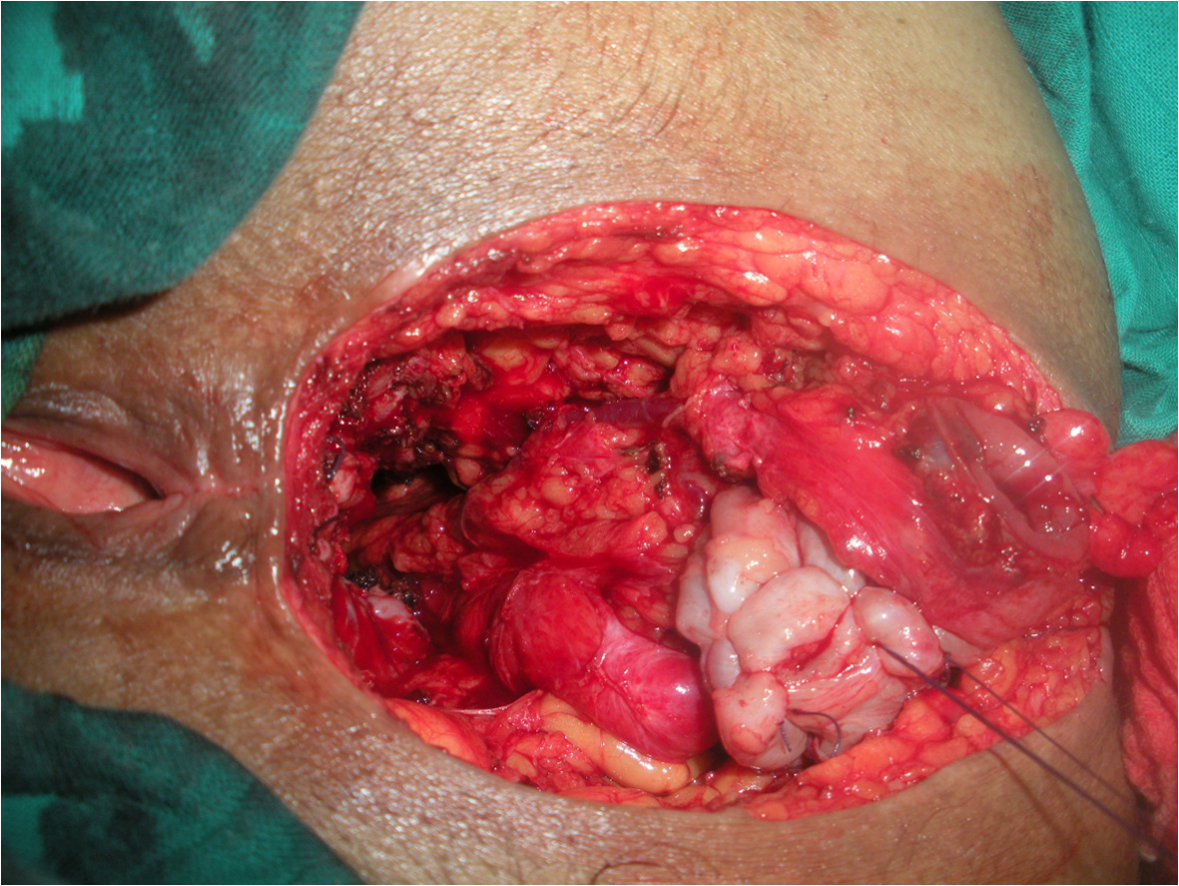
**Suture of the enrolled graft around the colon.**

**Figure 4:**
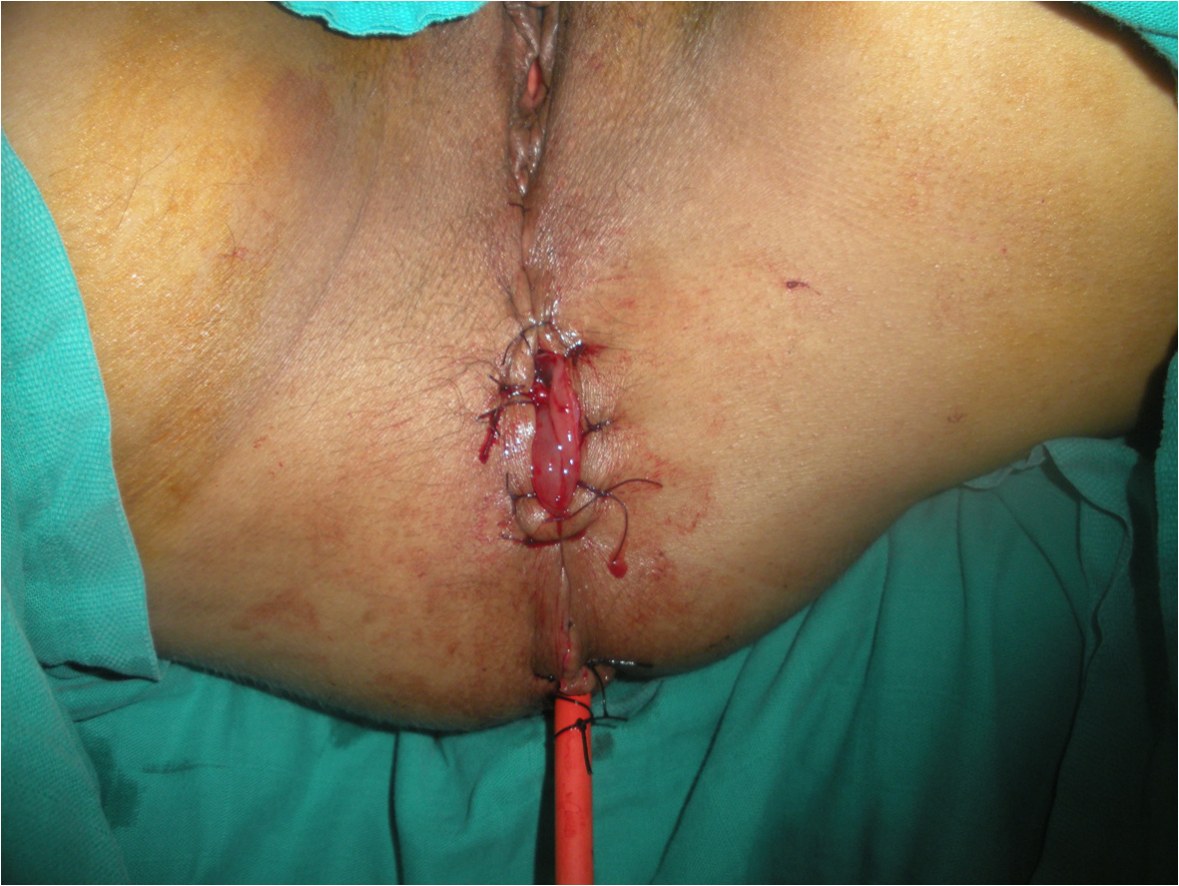
**Final aspect of the PCPC.**

Meticulous psychological preparation of patients and their families was achieved by explaining: surgical details, possible complications and risks of permanent stoma formation. The procedure is performed in two stages during the same intervention as a usual APR starting by a midline incision. Complete mobilisation of the splenic flexure and descending colon is required; a total excision of the mesorectum is accomplished reaching the level of the levator muscle; this step is ended by an omentoplasty based on the left gastroepiploic artery. Then the second step is done by a perineal approach ensuring an extended excision of the entire internal and external sphincter complex, allowing the excision of the specimen (Figure 1). Eight to ten cm of the end of the colon is resected and harvested as a free graft. This colonic fragment is stripped of its meso and epiploics (Figure 2a), then inverted and freed from its mucosa (Figure 2b) and placed in an antibiotic solution (Metrornidazol 500 mg) for 10 to 15 min. This graft is folded upon itself longitudinally keeping the serosal surface inside and wrapped snugly around the end of the colon 2–3 cm from its distal end for 1 and a half round. Absorbable 3.0 Sutures are taken to hold it in place (Figure 3). The end of the colon is brought out as a stoma in the perineum. The omentum is placed in the pelvis behind the colon. A drainage tube is placed in the perineum (Figure 4).

Colonic irrigations are started from the third day according to the protocol previously reported [7]. Patients and one of their family members are daily educated and assisted while performing colonic irrigations by specialized nurses.

### Assessment of oncological results and satisfaction score

Patients were first seen and examined by their own surgeons every 3 weeks till the complete healing of the wound. Then Follow-up, which included a clinical examination, a stoma examination and a liver ultrasound or abdomino-pelvic CT (alternately), was performed every three to 4 months for two years, every six months for three years and then once a year. A postoperative recurrence was stated by biopsy proven or radiographic evidence of recurrent disease. Survival was analysed according to Kaplan-Meier method using the IBM Corp. Released 2011. IBM SPSS Statistics for Macintosh, Version 20.0. Armonk, NY: IBM Corp. In all previous study of PCPC, Continence results were usually evaluated according to Kirwan continence scale [17]. Subjective satisfactory score was determined by asking patients to rate their satisfaction according to a scale from 0 to 10 (unsatisfied 0–3; moderately satisfied 4–7; satisfied 8–10) and to answer two open questions: Do you want to convert perineal colostomy to abdominal colostomy? If you had the choice again, would you choose PCPC or abdominal colostomy at primary intention?

## Results

There were 75 females and 71 males with a mean age of 47 ± 10 years. Details of population, tumour characteristic and postoperative outcomes are reported in Table 1.

**Table 1:** **Population characteristics**

**Characteristics**	**N (%)**
Gender	71 M/75 F
Mean age ± SD (years)	47 ± 10,4 (21–76)
CPC after	
Classic APR	114
Posterior pelvic exenteration	27
Total exenteration	2
In second step (after LQIC)	3
Preoperative radiotherapy	
• Alone (before 2000)	94 (64)
• Combined to chemotherapy	52 (36)
Tumour stage (UICC)	
I	22 (15%)
II	64 (43.8)
III	44 (30.1)
IV	8 (5.5)
Non precised	8 (5.5)
Postoperative mortality	0
Postoperative morbidity	24 (16.4)

UICC: international Union against cancer (2002) 6^th^ edition.

### Oncologic results

**Figure 5:**
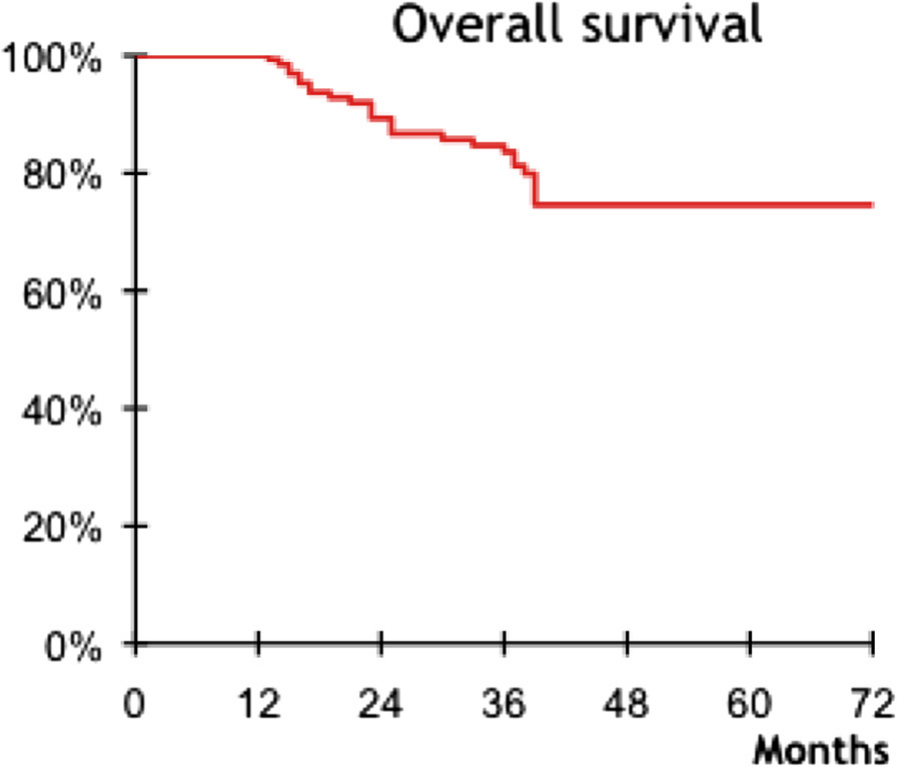
**Overall survival of 146 patients after APR and PCPC for low rectal adenocarcinoma.**

**Figure 6:**
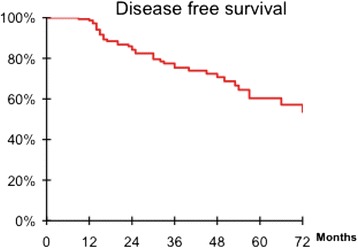
**Disease free survival of 146 patients after APR and PCPC for low rectal adenocarcinoma.**

TNM staging of rectal adenocarcinoma revealed stage I and II in 86 (59%) (Table 1). However, circumferential margin were not available for all patients the reason why they weren’t reported. After a median follow up of 36 months (range 12–156) months, the five-year overall survival and disease free survival rate were respectively 74,6% and 60,3% (Figures 5 and 6). Local and distant recurrences occurred respectively in 10 (6,8%) and 29 (20%) patients (Table 2).

**Table 2:** **details of satisfaction rate, functional and oncologicial results of 146 PCPC**

	**N (%)**
Kirwan stage of continence	
• A and B	100
• C, D and E	46
Satisfaction rate at the latest visit	
• Highly satisfied (8–10)	112 (77)
• Satisfied (4–7)	33 (22,4)
• Unsatisfied (0–3)	1 (0,6)
Oncological results	
• Median follow up of months	36 months (range 12–156)
• 5-year Overall survival rate	74,6%
• 5-year disease free survival	60,3%

All pelvic recurrences were diagnosed after digital examination of the perineal stoma. Median delay of local recurrences was 18,5 months (ranging from 13 to 66). Two patients had both pelvic recurrences and liver metastases and were treated by chemotherapy. All other patients had conversion to iliac colostomy after surgical excision of the recurrence except one who refused.

Distant Recurrences were treated with systemic chemotherapy. Liver metastases occurred in 20 (13,7%) patients. There were combined to lung and bone metastases respectively in 4 and one patient. Thirteen patients had only liver metastases and 5 of them were resected. Three (2,1%) patients had peritoneal carcinomatosis and one exclusive bone metastases treated by radiotherapy.

### Satisfaction rate

One hundred twelve (77%) patients were highly satisfied of this technique and 33 (22,4%) were moderately satisfied. Moreover, none of these patients accepted a conversion to abdominal colostomy even the unsatisfied 72 years woman (first open question). And as an answer to the second question: none of them would choose abdominal colostomy at first intent.

## Discussion

This study is the largest series of pseudocontinent perineal colostomy for rectal adenocarcinoma in the literature, showing that it is oncologically safe and provides high satisfaction rate.

Local and distant recurrences occurred in respectively 6,8% and 20% of the patients with an overall and disease free and five year survival rate of respectively 74,6% and 60,3%.

These results are comparable to classic APR with primary perineal closure in the literature [18-20], since PCPC is only a pelvic reconstruction after rectal resection respecting the rules of oncologic surgery. Moreover, the main theoretical oncological advantage of this technique is to allow an early diagnosis of pelvic recurrences by rectal examination or by echo-endoscopy [14,21], as the case of our study, all local recurrences were detected by clinical rectal inspection and confirmed by histological findings before starting adequate treatment.

Functional results of CPC are good with a high kirwan score (stage A and B) in 68% of the cases with no need to wear pads and no soiling [7]. More over, 77% of the patients were highly satisfied and none of them regretted the choice of this alternative to abdominal colostomy.

Many authors reported that permanent iliac colostomy could significantly alter patients’ quality of life by affecting negatively physical, sexual, social, and psychological aspects of life [8-10,22]. Cakmak and al demonstrated that social and sexual aspects of life are also affected in spouses of patients with colostomies [22]. Since 80% of patients reported that the reason for their inactive sexual life was their spouse’s abdominal colostomy, which they found repulsive [22]. Kuzu et al. showed that social, physical, sexual, and psychological aspects of life, in addition to religious worship, are severely impaired by sphincter sacrificing surgery in the Islamic population [11]. Indeed, daily praying and fasting were altered, since significantly greater number of Muslim who underwent APR stopped daily praying and did not fast during Ramadan [11]. In Islamic societies, religious rituals are considered as an important factor of social adaptation and improved quality of life.

In our study, we proposed the PCPC technique to our patients believing that it would be more adapted to their economic situation, social and religious specificities of our population. By allowing body image preservation, PCPC makes easy the social reinsertion and avoids the alteration of quality of life due to permanent iliac colostomy, especially in Muslim patients. These reasons could explain the high rate of satisfaction among our patients.

Several techniques of anal sphincter reconstruction and perineal closure have been described: myocutanus flaps as RAM flap to cover a large skin defect; artificial sphincter [23] or nearby skeletal muscles including gracilis, gluteus maximus or adductor longus muscle [24-26], with or without electrical stimulator transposed around the PCPC [27] to increase the continence of the neosphincter; more recently the combination of both RAM flap and PCPC [28] or . However, PCPC remains a simpler technique, easy to learn, that may be performed routinely after every APR in selected patients. It is performed by the same perineal incision, less costly to achieve with no need to apply any other synthetic material.

This study has some limitations, by its retrospective aspect and large period studied, we weren’t able to descibe more details about to pathological assessement, quality of mesorectal excition and lateral margins . Furthermore, our assessement of satisfaction was subjective and the use of QOL questionnary as (EORTC) would be more helpful. Further investigation related to specific impact of PCPC on muslim population should be done by comparing APR combined to abdominal colostomy to those with PCPC compared in two different groups according to their religions.

To our knowledge and by the time of writing this article, this is the largest study of patients who have undergone perineal colostomy for lower rectal adenocarcinoma that assessed the oncological results and subjective satisfaction rate of this technique. It allows good functional results without compromising oncological safety. Based on our data, we recommend the use of PCPC for perineal reconstruction in selected patients after APR for very low rectal adenocarcinoma, especially for Muslim patients.

## Conclusion

This study shows that pseudocontinent perineal colostomy is a simple, safe and reliable pelvic reconstruction technique after abdominoperineal resection for lower rectal adenocarcinoma. It provides a high degree of patient satisfaction without compromising oncological results. It is a good option in selected patients especially in the muslim and low-income countries population.
